# High-Throughput Analysis Reveals Stable Metabolome of *Paxillus involutus* Under White Light Exposure Despite Reduced Mycelium Growth

**DOI:** 10.3390/ijms27167405

**Published:** 2026-08-19

**Authors:** Łukasz Marczak, Aleksander Strugała, Agnieszka Szuba

**Affiliations:** 1 Institute of Bioorganic Chemistry, Polish Academy of Sciences, Noskowskiego 12/14, PL-61704 Poznań, Poland; lukasmar@ibch.poznan.pl (Ł.M.); astrugala@ibch.poznan.pl (A.S.); 2 Institute of Dendrology, Polish Academy of Sciences, Parkowa 5, PL-62035 Kórnik, Poland

**Keywords:** ectomycorrhizal fungi, GC-MS, metabolome, light exposure, soil fungi

## Abstract

Ectomycorrhizal fungi are rarely exposed to light in their natural soil habitat, and, in consequence, the metabolic effects of light exposure on mycelia’s molecular status remain largely uncharacterized. Meanwhile, such information may fill knowledge gaps and provide baseline data to understand their ecological resilience, abiotic stress responses, and metabolic regulation. This study analyzed the impact of white light on the metabolome of the ectomycorrhizal fungus *Paxillus involutus*. Mycelia were grown for six weeks under darkness (control) and white light conditions (150 μmol·m^−2^·s^−1^; 16/8 day/night periods). Treated mycelia were characterized by phenotypic alterations, mainly decreased growth and formation of more compact hyphal biomass. Although treated *Paxillus* had increased H_2_O_2_ concentrations, *P. involutus* exposed to light had unchanged malondialdehyde (MDA) levels, indicating that light exposure does not lead to severe oxidative stress. Indeed, a high-throughput GC-MS study revealed that the metabolome of mycelia exposed to light did not differ significantly from that of the controls, with only a few compounds showing altered abundances, all of which were more abundant in the light-exposed treatment. Lyxose and ribitol showed significant differences between treatments based on unadjusted *p*-values, as confirmed by individual *t*-tests, whereas only xylonic acid remained significant after FDR correction (α = 0.05). This finding is consistent with the overall pattern observed in the PCA and suggests that subtle changes in metabolite abundances may contribute to the observed phenotypic modifications. This first high-throughput metabolomic analysis of ectomycorrhizal mycelium exposed to light indicates that, although light is commonly considered a stress factor for soil fungi, it does not cause significant modifications in the *Paxillus involutus* metabolome, despite visible phenotypic changes in the mycelium. Although this study focuses solely on mycelium and does not investigate different light spectra, it may help explain potential implications for symbiotic plant interactions under fluctuating light conditions in soil ecosystems.

## 1. Introduction

Fungi represent one of the most diverse lineages of eukaryotic organisms, playing a fundamental role as decomposers, mutualists, or pathogens [1]. This enormous diversity of biological roles means that fungi differ significantly in their potential exposure to light. Plant pathogenic fungi, such as *Magnaporthe oryzae* and rust fungi, e.g., *Puccinia graminis*, which cause serious crop diseases by infecting living tissues, are often exposed to light and exhibit photoregulated virulence and developmental responses to light conditions [2,3]. Other fungal groups acting as endophytes can switch between commensalism and latent pathogenicity in plant hosts, with light conditions influencing fungal colonization and plant defenses under a complex interplay between their ecological roles [4,5]. Saprotrophic wood-decay fungi, such as *Pleurotus ostreatus* (oyster mushroom) or *Aspergillus nidulans,* colonize dead or dying trees to decompose lignocellulosic, decaying organic matter, especially plant debris and soil rich in nutrients; their mycelia and fruiting bodies develop on the exposed surface of logs or stumps where they are directly influenced by light [5,6]. Finally, mycorrhizal soil fungi occupy a specialized ecological niche by forming mutualistic associations with plant roots and by forming vast extra-radical mycelia belowground [7].

Light’s effects on fungal biology have been widely studied, with numerous reports on its impact on growth, metabolism, and differentiation. However, most research has focused on economically important, potentially light-exposed fungal species to identify effective control strategies [8,9]. The knowledge on the influence of light on mycelia functioning concerns, for the majority of fungal pathogens, species used in non-conventional medicine and in the food industry [10,11]; whereas, the influence of light on mycorrhizal fungi has been analyzed in the context of C acquisition of their plant partners, as it has long been known that arbuscular (AMF) and ectomycorrhizal fungi (ECMF) affect photosynthesis or directly influence the light compensation point of trees growing in symbiosis with them [12,13,14]. Unfortunately, although many (ecto)mycorrhizal species form big fruiting bodies at the soil surface [5,15], the effect of light on mycorrhizal mycelium remains largely unknown, especially at the metabolome level. This study addresses an existing knowledge gap.

Fungi are not photosynthetic, but they have photoreceptors that perceive light as an environmental signal. Fungi respond to visible light spanning blue to red wavelengths via three major photoreceptor systems: blue-light receptors (LOV-domain photoreceptors such as the fungal White Collar (WC) 1 protein and cryptochromes related to DNA-repair photolyases), red/far-red–sensing phytochromes, and membrane-bound rhodopsins that detect blue-green light through a retinal chromophore [16,17]. Genetic studies, namely reference genome sequence searches for photoreceptors in ECMF living underground, such as *Tuber* spp., suggest that they have rather a limited number of photoreceptors known from other trophic groups, most probably due to their light-deprived lifecycles. On the other hand, the presence of WC receptor genes indicates that EMCFs, even underground-fruiting, are likely to be functional and capable of responding to light [18,19].

In general, visible light is an important environmental signal for fungi. Fungi use light as a signal for the regulation of development, to guide the life cycle [20,21]. Light has been frequently reported, especially for economically important macrofungal species such as *Pleurotus ostreatus* L. and *Pleurotus eryngii*, to trigger the production of sexual reproductive structures [21,22,23,24]. Importantly, there are also reports demonstrating the effect of fluorescent light on the germination of *Glomus macrocarpum* propagules [25], confirming the significant role of light for mycorrhizal species.

Light, in particular, regulates gene expression and metabolic pathways, affecting cellulase activity, peptide and lipid metabolism, translation, and ribosome biogenesis, while also synchronizing the fungal circadian clock [8,16,21,23,26,27]. Moreover, it modulates the biosynthesis of secondary metabolites, including numerous mycotoxins, frequently in a wavelength-dependent manner [9,16,22,23].

Even for species living in ecological niches exposed to light, it is often perceived as a stressor [26]. The negative effects of light and UV radiation are frequently linked with ROS accumulation, macromolecules damage, heat-associated stresses, or desiccation. In response, fungi regulate light sensor function [21], alongside the modulation of stress-related genes in an attempt to enhance their tolerance [20,21]. Among the effective actions that prepare fungi for adverse conditions, the promotion of stress-related compounds like trehalose and erythritol, and biosynthesis of photo-protective pigments, carotenoids, or melanin, were reported [9,16,17,22,23]. Therefore, according to our hypothesis, in a species whose ecological niche is soil, we expect even stronger stress response to irradiance at the metabolome level, as light is not an element of their natural environment.

Metabolomics is a powerful tool among available phenotyping technologies, providing the ability to detect biological changes related to a particular factor versus analyzing numerous compounds simultaneously using high-throughput technologies [28]. High-throughput OMIC analyses were used previously to identify components secreted by free *Laccaria bicolor* mycelium, which may play an important role in interactions with plant partners [29,30] or to highlight the low temperature response in *Paxillus involutus* [31], but have been rather infrequently applied in studies of mycelium with respect to light-related effects. Among the few available studies examining metabolomic and transcriptomic responses during light-decreased beauvericin (BEA) biosynthesis in *Cordyceps chanhua* mycelium suggests that light promotes, in exchange, enhanced phenylalanine biosynthesis, likely redirecting metabolic flux toward pathways involved inter alia in previously mentioned pigment production [32].

In this study, we used a non-targeted high-throughput GC-MS approach to examine the metabolome of *Paxillus involutus* subjected to long-term exposure to white light.

*Paxillus involutus* (Boletales, Paxillaceae), selected for this study, forms ectomycorrhizal associations with a wide range of temperate and boreal tree species, including *Pinus*, *Picea*, *Betula*, *Fagus*, *Quercus*, *Populus*, and *Salix*, reflecting its broad host compatibility and ecological flexibility, facilitating forest nutrient exchange, soil structure formation and ecosystem stabilization, and contributing to forest productivity [15]. This important biological role makes *P. involutus* one of the most frequently analyzed species of ectomycorrhizal fungi, especially since its residual saprophytic properties [33,34] make it easy to establish new in vitro lines and cultivate in the lab [35,36]. Fungal ectomycorrhizal strains are usually cultivated in vitro in agar media in the dark (e.g., MMN medium; [37,38]). Moreover, during in vitro fungus–plant interaction experiments, hyphae are usually kept in the dark—one of the most frequently used procedures uses inoculated plant roots cultivated in Petri plates covered with aluminum foil to mimic natural conditions in soil [36]. However, light can penetrate several centimeters below the soil surface and may be an important factor for root and possible mycelial functioning, influencing, e.g., plant auxin metabolism and signaling [39]. Similarly, despite mycelia generally growing below the soil surface, light may influence fungal hyphae [18], e.g., during fruit body generation [40] or during in vitro experiments on ECM made in jars [38]. Therefore, a deep understanding of the impact of light on the functioning of *Paxillus involutus* free mycelium seems crucial.

## 2. Results

### 2.1. Phenotypic Changes and Basic Biochemical Features of Mycelia

**Figure 1:**
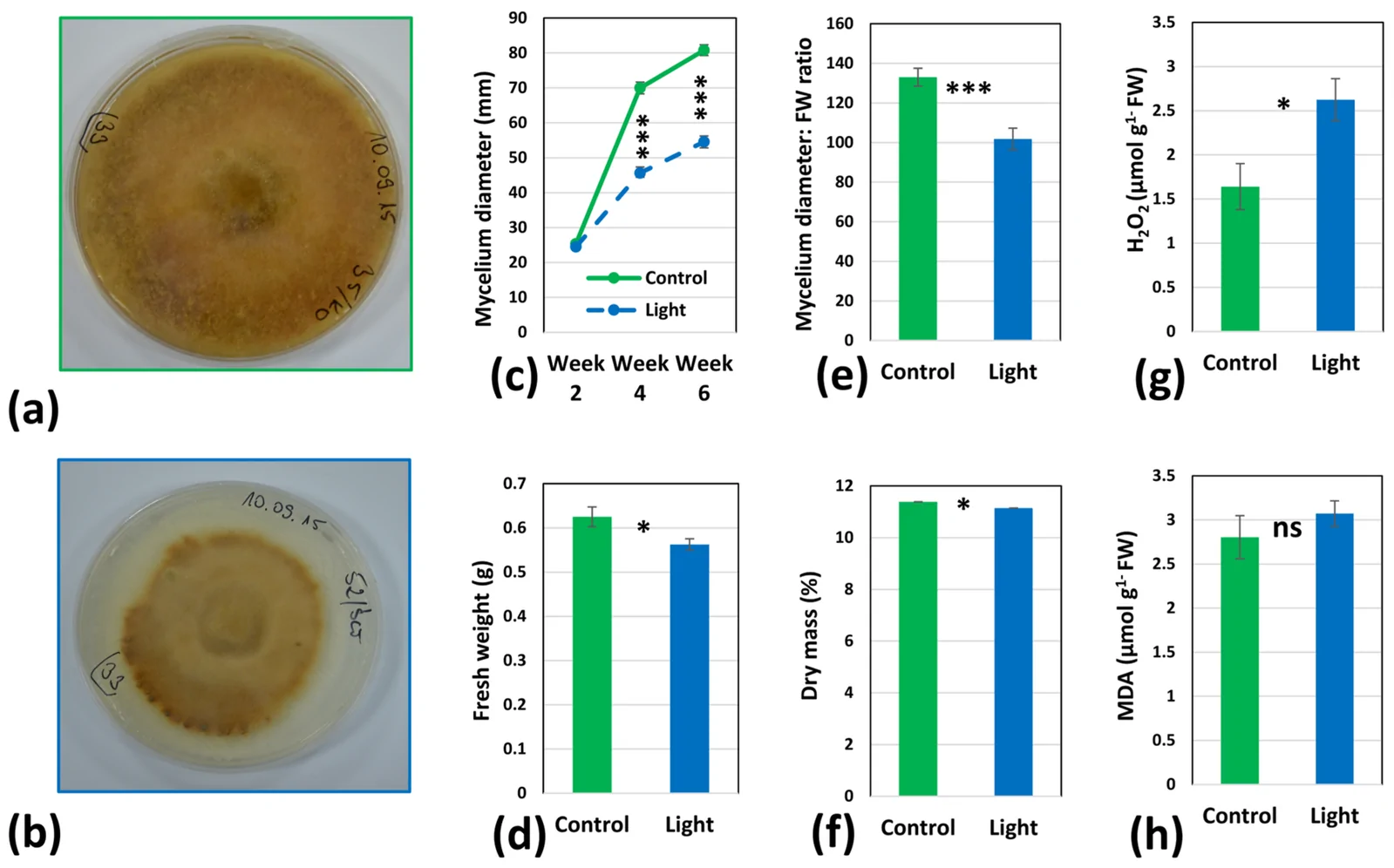
Treatment overview, biometrical and biochemical features of mycelia. Representative images of the control variant (**a**) and *P. involutus* exposed to light (**b**). Diameter, captured in 2nd, 4th, and 6th week of the experiment (**c**), fresh mass (**d**), and mycelium diameter (6th week): FW ratio (**e**). Dry matter percentage (**f**) of mycelia measured after six weeks of growth. Concentration of H_2_O_2_ (**g**) and concentration of MDA (**h**), both analyzed in *Paxillus involutus* mycelia after six weeks of growth and normalized with FW. For (**c**–**h**), mean values ± SE are presented. Significant differences according to the *t*-test (α = 0.05). * *p* < 0.05; *** *p* < 0.001, ns—not significant.

Light exposure caused a decrease in the radial growth (Figure 1a–c) and in mass of *Paxillus involutus* mycelia (Figure 1d). Additionally, the treated mycelium was more compact, what was reflected in its diameter: FW ratio (Figure 1e) but not in increased dry mass percentage, which was slightly, but significantly decreased in *Paxillus* exposed to light (Figure 1f). Agar pigmentations, reflecting compounds exuded into the growth medium did not differ between treatments, but tended to be darker in light exposed variants (*p* = 0.111; gray value detected for controls: 61.7 ± 3.6 (SE); and for light-exposed mycelium: 69.1 ± 2.5).

H_2_O_2_ concentration was higher in mycelia exposed to light, compared with controls (Figure 1g), whereas lipid peroxidation levels—here an indicator of macromolecule ROS-damage [31]—estimated based on MDA concentrations, did not differ between mycelia exposed to light and controls (Figure 1h).

Light exposure did not affect the carbon or nitrogen percentage in *P. involutus* mycelia (Table 1). Levels of numerous of analyzed elements also did not differ between controls and light-exposed mycelia, but potassium, sodium, and zinc were decreased in light-treated mycelia (Table 1).

**Figure 2:**
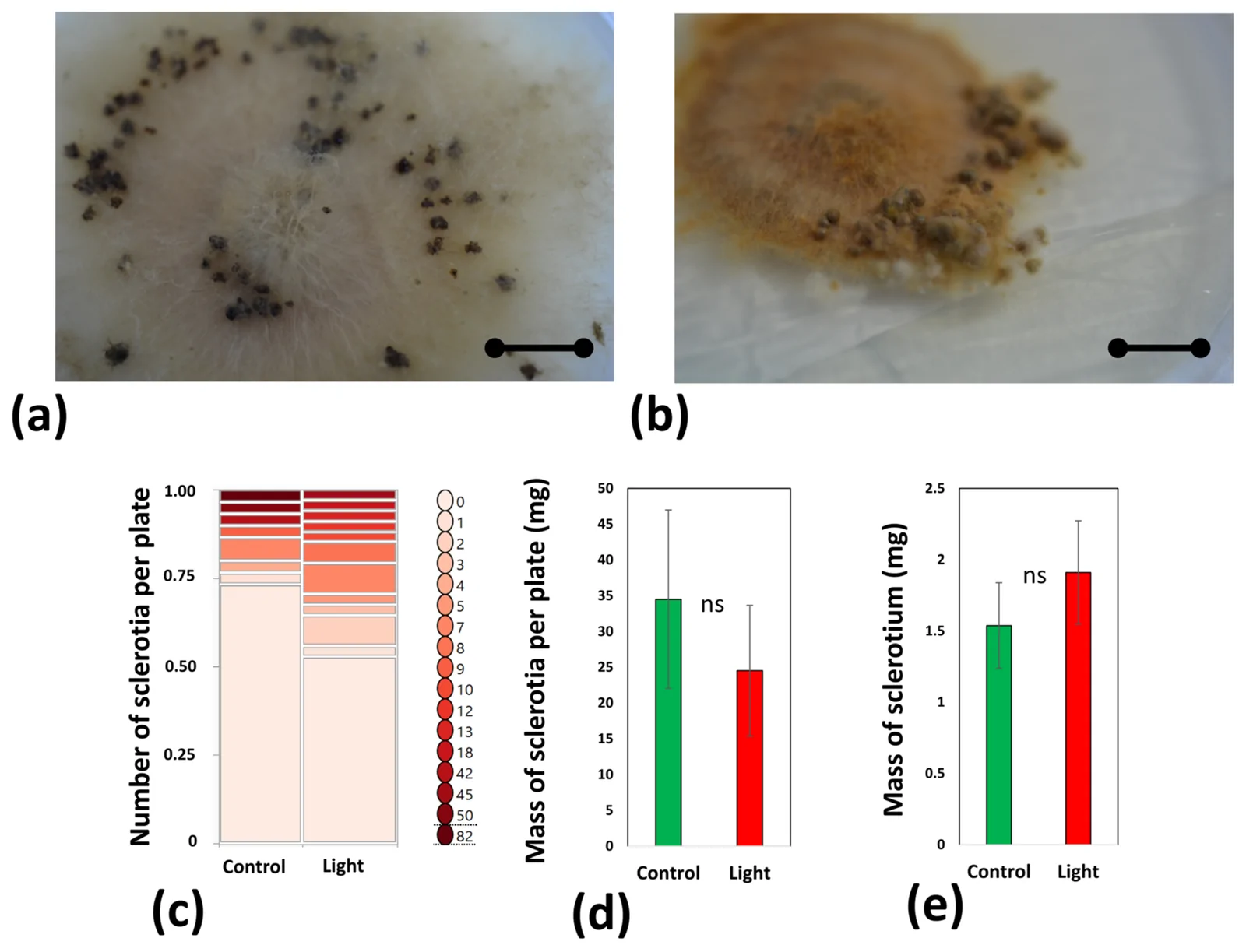
Sclerotial bodies. Representative image of sclerotia-producing mycelium growing under controlled conditions (**a**) and in exposure to light (**b**). Scale: 1 cm. The number of sclerotial bodies per plate, presented as a mosaic plot and illustrating the overall distribution of plates according to the presence and number of sclerotia detected. The size of each panel corresponds to the proportion of plates containing a given number of sclerotia, expressed as a percentage of the total number of analyzed plates (*n* = 12) (**c**). The total mass of all sclerotia detected per plate (**d**) and the mean mass of an individual sclerotium (**e**). For (**d**,**e**), mean values ± SE are presented. Significant differences according to the *t*-test (α = 0.05); ns—not significant.

In part of the analyzed trials, sclerotial bodies were recorded (Figure 2a,b). They occurred on a larger number of Petri dishes containing mycelium exposed to light, but the highest number of sclerotia per sample was recorded for the control variant (Figure 2c). Consequently, the average total weight of sclerotia per plate was (statistically insignificant) higher in *Paxillus involutus* cultivated in the dark (Figure 2d), despite the tendency to produce sclerotia with a higher weight in light-exposed mycelia (Figure 2e).

### 2.2. Paxillus involutus Metabolome (GC-MS Data)

**Figure 3:**
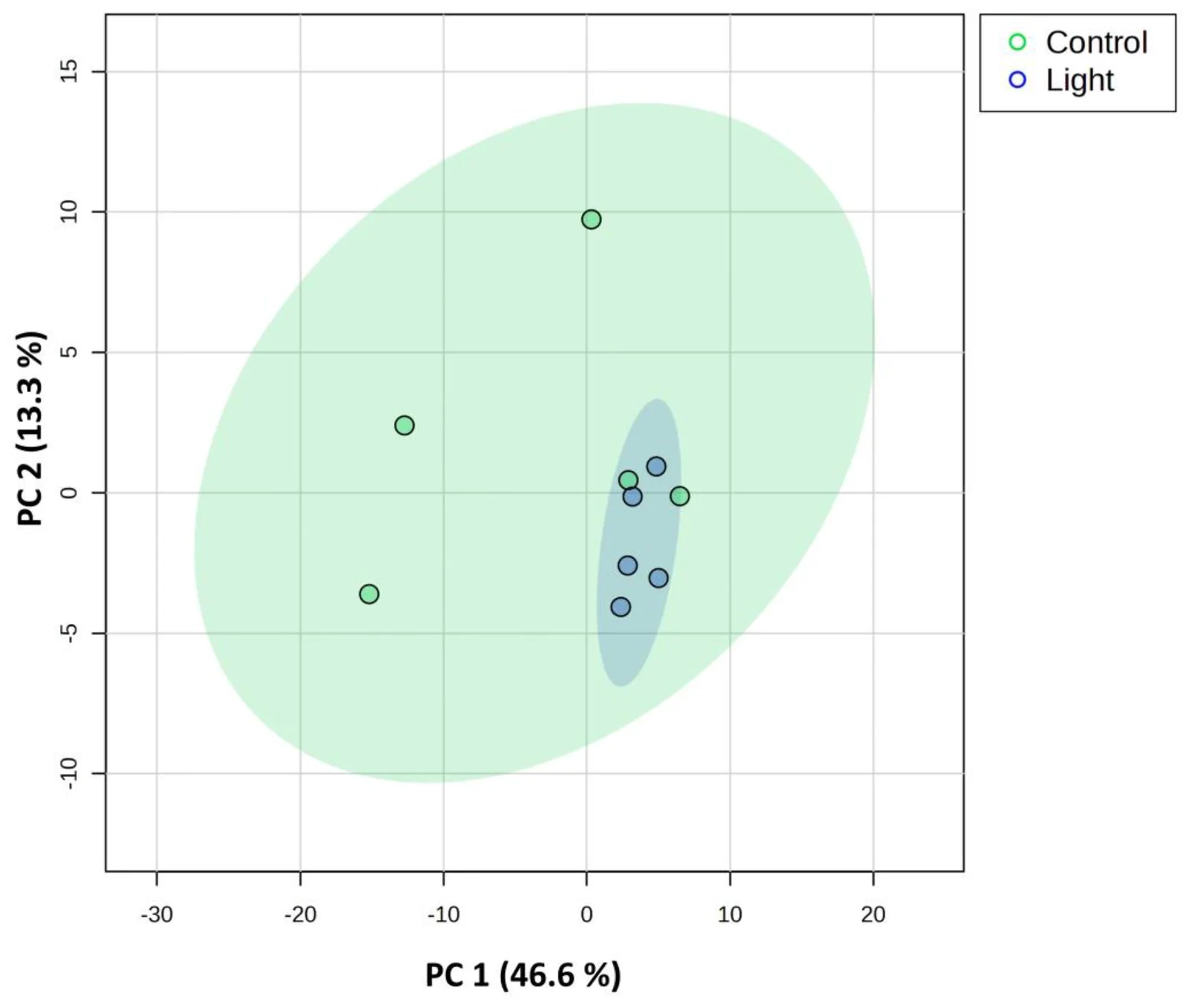
Principal Component Analysis (PCA) of metabolite composition detected in mycelium extracts (*n* = 5). Green points: control treatment, and blue points represent mycelia exposed to light. Ellipses represent the 95% confidence interval for each group.

Using a high-throughput GC-MS approach, we detected 123 metabolites in *Paxillus involutus* mycelia, among which amino acids and sugars, along with their derivatives and organic acids, were the most numerous (Supplementary File S1). According to the PCA, the metabolome of mycelia exposed to light showed substantial overlap with that of the controls, although some separation between the groups was apparent (Figure 3). Although the PLS-DA biplot showed more pronounced visual separation between the groups, cross-validation and permutation tests did not confirm this separation, supporting the substantial overlap observed in the PCA. All PLS-DA results, including the biplot, VIP compound analysis, and validation tests, are provided in Supplementary File S2.

**Figure 4:**
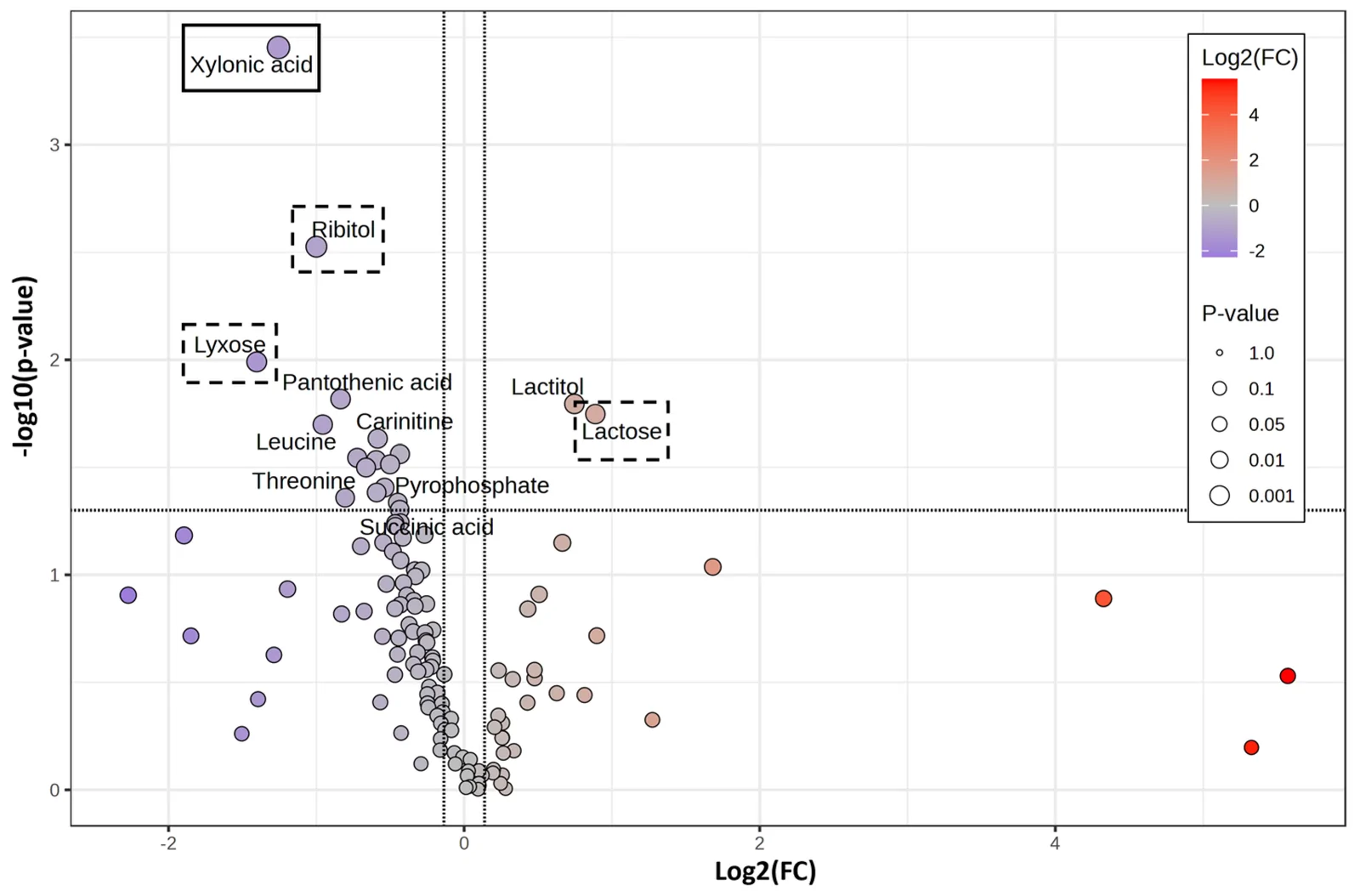
Volcano plots with determined compounds that were significantly different in abundance (α = 0.05) between analyzed treatments. Comparison between control treatment and compounds identified in the extract obtained from mycelia exposed to light. Purple points: compounds more abundant in light-exposed *Paxillus*, red points: more abundant in control. Solid-lined box—a single metabolite (xylonic acid) selected based on the FDR-adjusted *p*-value; dotted-lined boxes—metabolites showing differential abundance based on unadjusted *p*-values from the *t*-test, additionally tested individually using *t*-tests in JMP Pro 13.0.0 (for details see Supplementary File S2).

In agreement with PCA (Figure 3), very few compounds were detected as differently abundant (according to the *t*-tests; Figure 4) between the analyzed variants. Only lactose was lowered in its abundance in light-exposed *P. involutus*. Lactose, a glucose–galactose disaccharide not previously reported in *Paxillus* or other soil fungi, however, may reflect analytical ambiguity among structurally similar disaccharides and will not be further discussed.

The majority of detected compounds tended to be more abundant in light-exposed mycelia, with xylonic acid characterized by the most significant increase. Lyxose and ribitol were also significantly more abundant (based on unadjusted *p*-values; additionally confirmed by individual *t*-tests) (Figure 4).

These three metabolites, which were significantly enriched in light-exposed mycelia, also showed the highest VIP scores in the exploratory PLS-DA analysis (which, however, should be treated as exploratory; see Supplementary File S2 for details). In contrast, although numerous amino acid-related KEGG pathways tended to be enriched (with *p* < 0.05 but FDR > 0.05), only leucine and aspartate were detected among the top 10 VIP compounds (Supplementary File S2). In fact, we did not detect any significant enrichment of any of the metabolic pathways, but pathways involved in fructose and mannose, mentioned several amino acids, pantothenate, and CoA or nicotinate and nicotinamide metabolism, despite not meeting the FDR requirements, met the raw *p*-value criterion (*p* < 0.05; Supplementary File S2).

**Figure 5:**
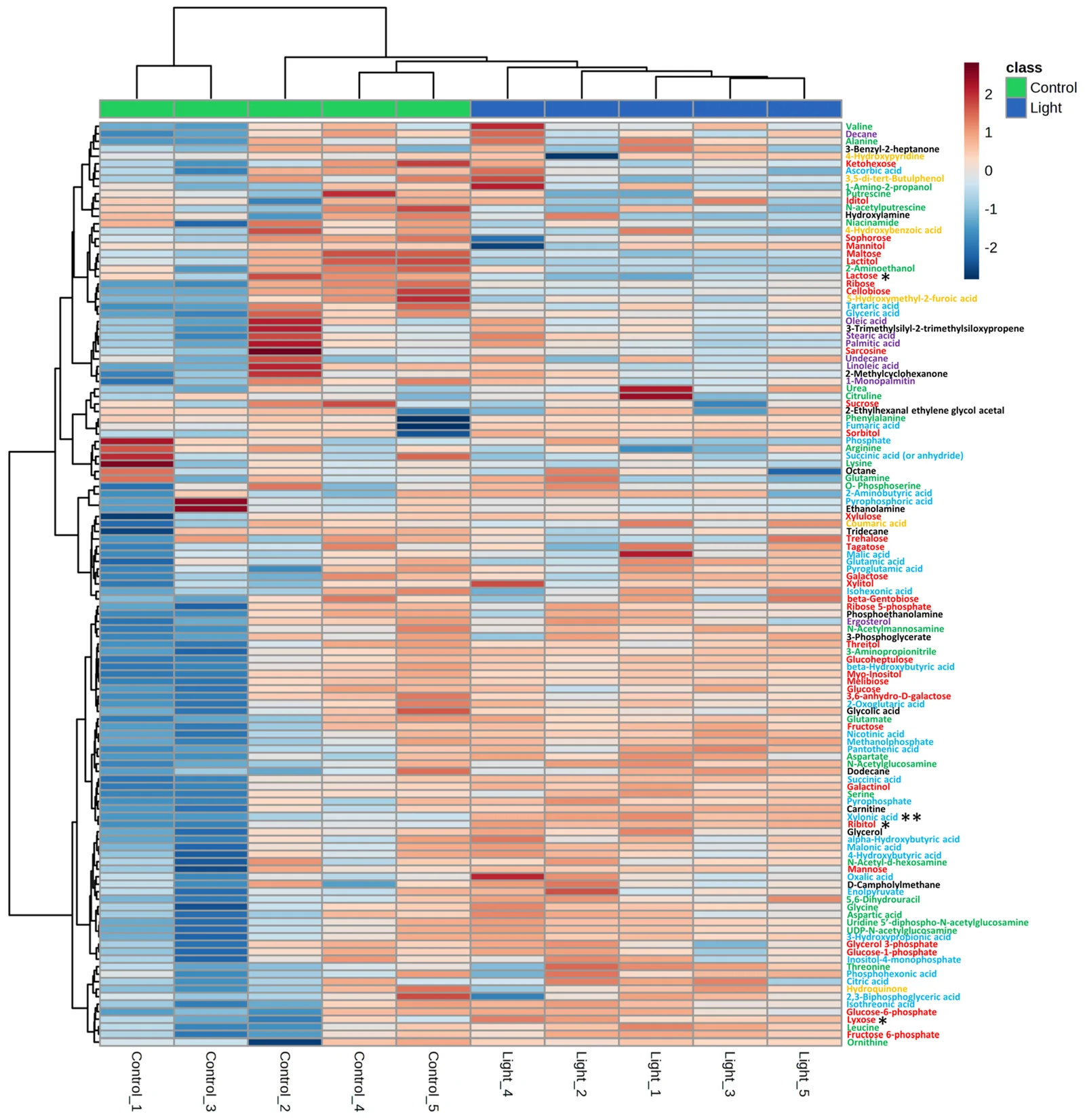
Heatmap analysis combined with hierarchical cluster analysis showing all detected metabolites. Intensity values were log 2-transformed, batch-corrected, and Z-scored rowwise. Blue, minimal abundance; red, maximal abundance. Compound names color code: blue—organic acids; green—amino acids and other main N-compounds; red—carbohydrates and their derivatives; purple—lipidic compounds; orange—phenolic compounds; black—remaining compounds (Supplementary File S1). **—compound selected based on corrected multiple *t*-test; *—compounds selected based on a raw multiple *t*-test, whose significance was confirmed using JMP Pro 13.0.0 software.

Apart from a small number of significantly alerted compounds, light exposure did not induce clear trends (changes in the abundance below the defined cut-off threshold) for the major groups of metabolites, as shown by the heatmap analysis including all identified compounds to reveal broader trends in the metabolome beyond statistically significant changes (Figure 5). Neither sugars, nitrogen-containing compounds, nor lipids showed a consistent tendency toward either decreases or increases in their abundances. Nevertheless, trials from individual variants are grouped, although without full coverage with hierarchical analysis (Figure 5).

In summary, white light affects *Paxillus involutus*; our results indicate that it does not induce global, large-scale reprogramming of the mycelium metabolome.

## 3. Discussion

### White Light Exposure—Effect on the P. involutus Metabolome Lower than Expected

Light is considered one of the important environmental factors influencing fungal life. It is known that even belowground, ectomycorrhizal fungi have various chromophores [18], but still the impact of light on ectomycorrhizal fungi is analyzed primarily in the context of their influence on light acquisition by plant hosts, e.g., ref. [41]. Consequently, this report presents the first high-throughput GC-MS analysis of the effect of light on a pure culture of ectomycorrhizal mycelium. The distinct ecological niche of ECMF may impose different selective pressures than those experienced by saprotrophic fungi, whose responses to light have been more extensively studied [8,21,23,27,40] potentially resulting in distinct light-response strategies.

Due to the specific ecological niche of the soil fungi, light is generally regarded as a stress factor [26]. It was reported that light negatively affects the biomass of soil fungi [42], which we also detected in light-exposed *Paxillus involutus*.

The reduced growth represents a typical fungal response to various stressful conditions, e.g., refs. [31,43]. Such a response is frequently linked to disruptions in cellular respiration, leading to reduced energy supply or energy redistribution, where resources are allocated to redox homeostasis and stress protection rather than hyphal proliferation [44,45]. Concomitantly, the biosynthesis of key structural components may be downregulated, while metabolic fluxes are usually redirected toward the increased production of protective metabolites, including osmoprotectants, antioxidants like proline, lysine, phenylalanine, tyrosine, or γ-aminobutyric acid [13,31,46,47,48]. Visible light modulates stress-related genes in various fungi, enhancing tolerance to heat, osmotic, and oxidative stresses by inducing protective HSP proteins, stabilizing metabolites such as trehalose, and promoting compounds like erythritol or mannitol, effectively acting as an environmental alert system that prepares fungi for adverse conditions [17,49,50].

It should be noted that the GC-MS method does not allow the detection of certain anti-stress compounds. On one hand, enzymatic antioxidant systems (e.g., SOD, CAT) may be upregulated at the protein/transcript level, requiring no substantial metabolic adjustment. On the other hand, the detection of core antioxidants such as glutathione and ascorbic acid is not covered due to polarity and derivatization efficiency constraints, so activation of the antioxidant system cannot be completely ruled out. However, it is interesting that in ectomycorrhizal *P. involutus* exposed to light, we did not observe increases in known anti-stress compounds; in fact, trehalose, a well-known fungal anti-stress compound, tended to be decreased in light-exposed *Paxillus* (Supplementary File S1). In our study, we did not detect alterations in saturated fatty acids or other lipids, unlike those reported in light-exposed *Neurospora crassa* and *Pleurotus eryngii* [26,51]. Light intensity or even wavelength changes can influence phenolic compound abundances in fungi growing in ecological niches exposed to light [52,53]; however, the shikimate acid pathway was not activated in *Paxillus*, and only a few phenolics, known anti-stress compounds, were detected in the analyzed mycelia (Supplementary File S1).

Resources may potentially be diverted from vegetative growth toward reproductive or resting structures rather than invested in extensive metabolic stress defense, and, indeed, sclerotial formation has previously been associated with reduced mycelial growth (e.g., ref. [54]). Sclerotial production in *Paxillus* has been suggested to be induced by abiotic stress, although the evidence remains ambiguous [31,55], and the effect of light has not yet been investigated. In other fungi, including *Sclerotium rolfsii* and *Flammulina velutipes*, light can promote sclerotial formation (e.g., ref. [48]). Our results suggest a possible increase in sclerotial size under light, although only a trend was observed. The lack of significant light-induced sclerotial body formation or metabolic evidence of resource allocation toward these structures in *P. involutus* argues against the hypothesis that preferential investment in sclerotia underlies both the observed growth inhibition and the limited metabolic stress response. Further studies are needed to test this hypothesis.

In the mycelium we studied, light caused a slight (insignificant) tendency to release dark-colored compounds into the growth medium. Stimulation of the production of melanin and other UV-protecting pigments has been repeatedly demonstrated under the influence of light [18,56]. No melanin has been detected in *Paxillus* to date, although it is detected in ectomycorrhizal fungi [57]. However, in *Paxillus*, the role of melanin is partially taken over by pigments characteristic of the family, such as involutin [58], but at the same time, involutin is mainly produced during the Fenton reaction. A major part of the involutin produced by *P. involutus* during organic matter decomposition was secreted into the medium, and the metabolite was not usually detected/analyzed when the fungus was grown on a mineral nutrient medium [58]. In addition, the protective role of involutin against UV light, one of the main roles of melanin in fungi, has not been proven [59], which may explain the lack of statistically significant changes in mycelium pigmentation.

Exposure of *Paxillus involutus* to light resulted in very limited metabolomic changes, restricted to xylonic acid, lyxose, and ribitol. Xylonic acid and lyxose are pentose metabolites linked to the pentose phosphate pathway (PPP), which provides NADPH for redox balance and precursors for nucleotide and amino acid biosynthesis. Changes in these pentose-related metabolites may potentially indicate PPP rewiring to support oxidative stress defense, as H_2_O_2_ increased without elevating MDA [26,60,61]. However, H_2_O_2_ may also act as a signaling molecule involved in the regulation of mycelium development in *Paxillus involutus* [62], potentially contributing to the observed denser hyphae and sclerotial formation. *Paxillus involutus* also employs H_2_O_2_ in controlled oxidative processes, including Fenton-based organic matter decomposition [58]. Moreover, H_2_O_2_ has been shown to participate in physiological processes in the *P. involutus*–plant system, supporting its potential role beyond that of a mere oxidative damage marker [62].

Ribitol, a polyol, may function as a compatible solute, buffering redox state and stabilizing cellular structures [63]. Its selective accumulation under light may indicate enhanced polyol biosynthesis associated with oxidative or osmotic stress responses in fungi [64,65].

Overall, the observed changes point to selective metabolic adjustments linked most probably to redox balancing and stress adaptation rather than broad metabolomic reprogramming. This picture, revealed by metabolome analysis, suggests a lack of global reprogramming of the mycelium metabolome, which is nevertheless surprising in combination with a significant reduction in mycelium growth. Our results may indicate that fungal phenotypic changes (e.g., growth reduction) do not necessarily translate into large-scale steady-state metabolome changes, as fungi respond to environmental or growth perturbations via selective, pathway-specific metabolic adjustments, while core intracellular metabolite pools could remain relatively stable.

Moreover, even in processes strongly influenced by light, such as conidia production, the effects of illumination were not always consistent, with substantial differences observed not only between species but also among fungal strains [53,66].

Anti-stress compounds not being upregulated in fungal cells exposed to light may indicate the absence of severe stress, although other abiotic stressors have elicited pronounced responses in *Paxillus involutus* [46,62]. The low-level oxidative stress observed by us may also be related to the long period of exposure to the stress: fungal cells were not exposed to acute stress, but grown constantly under unfavorable conditions, probably at least partially acclimatized to it [31]. Constant exposure to light induces a transient transcriptional response in fungi that leads to photoadaptation [17,67]. The subtle metabolic changes might therefore reflect precise adaptive regulation, potentially associated with energy conservation strategies, rather than metabolic dysfunction, as none of the central metabolic intermediates was significantly affected. Nevertheless, the limited metabolic response to light appears to be distinct, as similarly long exposure to low temperature, for example, triggered significant alterations in the mycelial N-metabolome of *Paxillus* [31]. In various fungal species, light response and photoadaptation are influenced by multiple environmental factors, including nitrogen and phosphorus availability, but are particularly sensitive to carbon-glucose deficiency [23,27,31,68,69]. In our study, however, no carbon limitation was imposed, and carbon and nitrogen (%) did not differ between the analyzed mycelia. The stable carbon level may additionally explain, at least in part, the absence of substantial changes in the metabolome of light-exposed *Paxillus*, as there was no additional factor triggering an enhanced light-exposure effect in other fungal species.

In fruiting species, light is often a signal that initiates or modulates the development of reproductive structures. Exposure to different wavelengths alters the expression of many genes, for example, those involved in hyphal knot formation and mycelium branching towards fruiting body development in other fungal species [68,70], which causes an increase in hyphal density, an effect observed in *P. involutus* exposed to light. In fact, the role of light in *Basidiomycota* focuses on regulating spore induction, asexual/sexual reproduction, and fruit body maturation [18]. This fact suggests that the lack of *Paxillus* metabolome response to light exposure may also have another source.

The in vitro line used in the study was derived from the fruiting body [38], a reproductive organoid of *Paxillus involutus* exposed to light [15]. Comparative studies of mycelium versus fruiting bodies in numerous species have revealed phenotypic and molecular differences resulting from various expression patterns and molecular states [69]. These differences may affect carbohydrate metabolism, cell wall structure, pigmentation, reproduction, and specific functions of the fruiting body [71,72,73]. These studies focused on transcriptomics, but differences were also demonstrated between polysaccharides (e.g., various β-glucans), proteins associated with defense and metabolism, and degradation enzymes [26]. Metabolomic changes are therefore different in fruiting bodies (from which the analyzed in vitro line originates) compared to extramatrical mycelium, which in nature functions in dark conditions [74]. It cannot, therefore, be ruled out that light has a significant negative effect on mycelial cells functioning in the dark, though not necessarily on the basic vegetative metabolism of mycelium derived from a fruiting body naturally exposed to light.

In summary, despite phenotypic alterations, the metabolome of *Paxillus involutus* mycelium undergoes only minor changes when exposed to light, showing only trends that partially overlap with light-induced changes observed in other fungal species. Contrary to our hypothesis, the results indicate a lack of a strong anti-stress response in the light-exposed mycelium. This effect may be related to the chronic nature of the applied treatment, the origin of mycelium from a light-exposed fruiting body, or to the absence of other factors potentially enhancing the light effect (such as changes in carbon availability), but further research is needed to confirm those hypotheses. Importantly, the present study provides only an endpoint snapshot after six weeks of light exposure. Future research combining time-resolved sampling with short-term and long-term light treatments could help distinguish acute stress responses from photoadaptation and determine whether the observed metabolomic stability represents an inherent feature of *Paxillus involutus* or an acclimation response. Extending these investigations to the *P. involutus*–plant symbiosis could provide further insight into how fungal light responses may translate into functional consequences for the mycorrhizal association.

## 4. Material and Methods

### 4.1. Fungal Culture and Treatments

The *Paxillus involutus* strain, obtained from a fruit body growing in a poplar monoculture, was barcoded, introduced into an in vitro culture, and maintained in Petri dishes in a Modified Melin-Norkrans agar medium (MMN; [75]). The *P. involutus* genotype analyzed in this study was previously tested in our lab in the context of ectomycorrhizal interaction with *Populus × canescens* [13,38] or low temperature response [31]. In the present experiment, 5 × 5 mm fragments of fresh mycelia were transferred to Petri dishes with an agar medium covered with cellophane. Mycelia grew either in controlled conditions (in full darkness at 21 °C (±1 °C); treatment ‘Control’), or under a 16 h/8 h day/night photoperiod using a cool white fluorescent lamps (Sylvania BriteGro F36W/T8/2084; 36 W; spectral range 400–700 nm) at a photosynthetic photon flux density (PPFD) of 150 μmol m^−2^ s^−1^ throughout the whole experiment (treatment ‘Light’). Light was the sole variable between the two groups.

The diameter of mycelia was measured from high-resolution images captured at 2, 4, and 6 weeks of growth using ImageJ v1.48 software (Wayne Rasband, Bethesda, MD, USA) (*n* = 12). After six weeks of growth, the mycelia were harvested, weighed, and stored at −80 °C until analyzed. The mycelium was collected whilst exposed to light. Additionally, the sclerotia produced by the mycelium were counted and weighed. The pigmentation of the agar growth medium was additionally assessed by determining the maximum gray values from images of the agar plates captured at the end of the experiment and then converted into the gray scale (using ImageJ software).

### 4.2. Mineral Analysis

Samples of mycelia dried at 60 °C for 48 h (*n* = 12) were ground into a powder in a ball mill, mineralized, and analyzed to determine their concentrations of Mg, K, Ca, Na, Fe, Co, Cu, and Zn. The samples were analyzed in a microwave mineralizer (Multiwave 3000, Anton Paar, GmbH, Graz, Austria) and an inductively coupled plasma time-of-flight (TOF) mass spectrometer (Spectrometer OptiMass 9500 ICP-TOF-MS, GBC Scientific Equipment, Hampshire, Braeside, Victoria, Australia) owned by the Institute of Dendrology, Polish Academy of Sciences, Kórnik, Poland. For all runs, the certified reference material (NCS DC 73349 Bush Branches and Leaves; China National Analysis Center for Iron & Steel, Beijing, China) was used. Total nitrogen and carbon were determined in dried and ground mycelia (*n* = 3) with a CHNS analyzer (2400 CHNS/O Series II System, PerkinElmer, Waltham, MA, USA), after which the C/N ratio was calculated.

### 4.3. MDA and H_2_O_2_

H_2_O_2_ content was tested according to Zhou et al. [76]. The fungal hyphae (100 mg samples; *n* = 6) were homogenized at 4 °C with activated carbon and 5% trichloroacetic acid (TCA) at the ratio of 1: 0.2: 1 (*w*/*w*/*v*). H_2_O_2_ concentration was determined using a colorimetric reagent made by mixing 0.6 mM 4-(2-pyridiazole) resorcinol disodium salt with 0.6 mM titanium potassium oxalate at a 1:1 ratio in 50 mM phosphate buffer (pH 8.4); absorption was measured at λ = 508 nm. The concentration of hydrogen peroxide was read from a standard curve and was reported as μmol per g fresh mycelium (μmol g^−1^ FW).

To estimate the intensity of lipid peroxidation in *P. involutus* hyphae, we determined malondialdehyde (MDA; *n* = 6) concentration in frozen mycelia homogenized in 0.25 M H_2_SO_4_/10% TCA solution according to modified methods of Shah et al. [77]. Thiobarbituric acid (TBA) was added to the extract mixture (to make 0.25% of the mixture’s vol.). After 10 min of boiling, the reaction of TBA with MDA was stopped by cooling (on ice). The supernatants were analyzed at 532 nm to determine the reaction products and at 600 nm for nonspecific reaction products, calculated per g FW [78].

### 4.4. Metabolome Study

Metabolites for GC-MS were isolated from 20 mg samples of mycelia (*n* = 5) by methanol extraction and were further derivatized using a standard procedure. The extracts were analyzed with an Agilent 7890A gas chromatograph coupled to a Pegasus 4D GCxGC-TOFMS mass spectrometer (LECO, St. Joseph, MI, USA). The compounds were separated using a DB-5T bonded-phase fused-silica capillary column (30 m length, 0.25 mm inner diameter, 0.25 μm film thickness) (J&W Scientific Co., Folsom, CA, USA). The GC oven temperature program was as follows: 2 min at 70 °C, raised by 10 °C/min to 300 °C, and held for 10 min at 300 °C. The GC analysis lasted a total of 36 min. Helium was used as the carrier gas at a flow rate of 1 mL/min. One microliter of each sample was injected in splitless mode. The initial injector temperature was 40 °C for 0.1 min, after which the temperature was raised by 6 °C/min to 350 °C. The septum purge flow rate was 3 mL/min, and the purge was turned on after 60 s. The transfer line and ion source temperatures were set to 250 °C. In-source fragmentation was performed at 70 eV. Mass spectra were recorded in the range 35–850 *m*/*z*. Data acquisition, automatic peak detection, mass spectrum deconvolution, retention index calculation, and library searches were performed using LECO ChromaTOF software, version 4.51.6.0 (LECO Corporation, St. Joseph, MI, USA). To eliminate retention time (Rt) shift and determine retention indexes (RI) for each compound, an alkane series mixture (C-10 to C-36) was injected into the GC-MS system. The metabolites were identified automatically with a library search (NIST library); an analyte was considered identified when it passed a quality threshold, i.e., a similarity index (SI) above 700 and a matching retention index ± 10. Artifacts (alkanes, column bleed, plasticizers, MSTFA, and reagents) were identified using the same method and then excluded from further analyses. To obtain accurate peak areas for the deconvoluted components, unique quantification masses for each component were specified, and the samples were reprocessed. In the resulting data tables (the ion intensities of the multiple identifications were summed up; the raw and final data tables are presented in the Supplementary File S1). Pooled quality control (QC) samples were included in the analytical sequence to monitor the stability and reproducibility of the GC-MS measurements. Further analyses were performed using the MetaboAnalyst 6.0 platform (https://www.metaboanalyst.ca/; accessed on 14 and 25 November 2025). The obtained profiles were normalized against the sum of the chromatographic peak areas (using the TIC approach) as a global normalization procedure for comparing relative metabolite abundances among samples, auto-scaled, and used for further analysis. Pathway enrichment was performed using the default MetaboAnalyst settings and the *Saccharomyces cerevisiae* (yeast) KEGG database as a well-characterized fungal reference, as *Paxillus involutus* is not available in MetaboAnalyst. Therefore, the results were considered exploratory and interpreted as potentially conserved fungal metabolic pathways rather than species-specific pathway activity. Quantitative metabolite set enrichment analysis was performed against the KEGG library (Supplementary File S2).

### 4.5. Statistics

Biometrical and biochemical mycelial data were analyzed using JMP Pro 13.0.0 software (SAS Institute Inc., Cary, NC, USA). Values were considered significant at *p* < 0.05 as determined with *t*-tests. The ion intensities were transformed to log values, auto-scaled, and all samples were grouped using a categorical annotation. The values were filtered for blanks in the samples using the default MetaboAnalyst settings, and PCA and PLS-DA were performed on the resulting dataset. A significance threshold of α = 0.05 was used for *t*-tests, FDR = 0.05 for multiple-testing correction, and, given the small differences observed in the PCA, a relatively lenient FC cut-off of >1.1 for the Volcano plot analysis. Whereas clustering analysis of all detected metabolites was performed on data normalized using the Z-score algorithm and presented on the heatmap. Additionally, the initial intensities of metabolite ions, which were selected based on raw *p*-values (<0.05), were analyzed (*t*-test) using JMP Pro 13.0.0 software (Supplementary File S2).

## Figures and Tables

**Table 1:** Concentrations of elements in *Paxillus involutus* mycelia. Results are presented as the mean value ± standard error of twelve independent replicates (for C(%) and N(%) for three replicates). Significant differences between analyzed treatments are highlighted in bold (*t*-test; α = 0.05).

Element	Control	Light	*p* Value
C (%)	44.17 ± 0.28	44.72 ± 0.24	0.206
N (%)	2.16 ± 0.03	2.083 ± 0.11	0.5126
C/N	23.81 ± 0.22	25.17 ± 1.21	0.333
Mg (µg g^−1^)	2243 ± 23	2389 ±101	0.171
P (µg g^−1^)	11,973 ± 196	12,282 ± 607	0.633
K (µg g^−1^)	21,013 ± 352	18,733 ± 872	**0.024**
Ca (µg g^−1^)	799 ± 34	737 ± 42	0.263
Na (µg g^−1^)	210.6 ± 6.0	190.5 ± 4.7	**0.014**
Fe (µg g^−1^)	630.7 ± 26.1	647.1 ± 8.7	0.557
Cu (µg g^−1^)	20.02 ± 0.26	18.75 ± 0.59	0.061
Zn (µg g^−1^)	55.73 ± 14.17	24.16 ± 21.22	**0.037**

## Data Availability

The original contributions presented in this study are included in the article/Supplementary Materials. Further inquiries can be directed to the corresponding author.

## Supplementary Materials

The following supporting information can be downloaded at: https://www.mdpi.com/article/10.3390/ijms27167405/s1.
